# Clinical efficacy and biomechanical analysis of 3D-printed guide plate–assisted cannulated screw fixation compared with conventional fixation in complex acetabular fractures

**DOI:** 10.3389/fsurg.2026.1797007

**Published:** 2026-07-15

**Authors:** Chaoqiang Wang, Xiaoyu He, Zhongyu Zhang, Xueli Li, Mei Wang, Feng Zeng, Caosheng Lai, Huimei Li, Peng Chen, Zhiping Zhou

**Affiliations:** 1 Department of Sports Medicine, The First Affiliated Hospital of Fujian Medical University, Fuzhou, Fujian, China; 2 Department of Orthopedics, Mindong Hospital Affiliated to Fujian Medical University, Fu’an, Fujian, China; 3 Department of Dermatology, Mindong Hospital Affiliated to Fujian Medical University, Fu'an, Fujian, China

**Keywords:** acetabular fractures, bone screws, finite element analysis, internal fixation, three-dimensional printing

## Abstract

**Background:**

Optimal fixation for pelvic and acetabular fractures remains challenging due to competing demands for stability and minimally invasive surgery. This study combined finite element (FE) modeling and a retrospective clinical analysis to compare three fixation strategies.

**Methods:**

A three-dimensional FE model of a representative both-column acetabular fracture was constructed to compare cannulated screw-only, plate-only, and combined cannulated screw–plate fixation under simulated standing and sitting conditions. Clinically, patients with acetabular fractures treated between November 2021 and May 2023 were retrospectively grouped into 3D-printed guide plate–assisted cannulated screw plus plate (*n* = 8), conventional cannulated screw plus plate (*n* = 14), and plate-only fixation (*n* = 12). Perioperative outcomes, postoperative complications, and 3-month hip function were analyzed with multivariable adjustment.

**Results:**

FE analysis showed higher stress and displacement under sitting than standing across all constructs, with combined cannulated screw–plate fixation providing better fracture-line displacement control than screw-only fixation, particularly under sitting conditions. Clinically, after multivariable adjustment, the 3D-printed guide group had significantly shorter operative time than the plate-only group and the conventional screw plus plate group (−56.15 min, *P* = 0.048; −68.46 min, *P* = 0.031, respectively), lower intraoperative blood loss than the plate-only group (−575.48 mL, *P* = 0.001), and shorter incision length than the plate-only group (−7.94 cm, *P* = 0.004). Both the 3D-printed guide group and the conventional screw plus plate group showed significantly lower total hospitalization costs than the plate-only group (−39,944.29 RMB, *P* = 0.040; −32,527.55 RMB, *P* = 0.048, respectively). No significant adjusted differences were observed in complication risk, 3-month hip function, or hospital length of stay.

**Conclusions:**

Combined cannulated screw–plate fixation provides superior biomechanical stability for complex acetabular fractures, while patient-specific 3D-printed guide plate significantly improve perioperative efficiency without compromising short-term safety or functional recovery.

## Introduction

Pelvic and acetabular fractures are uncommon but clinically severe injuries, typically resulting from high-energy trauma and associated with substantial morbidity, prolonged hospitalization, and increased mortality. Epidemiological studies indicate that pelvic or acetabular fractures account for approximately 3%–8% of all fractures, with reported mortality rates ranging from 4% to 28% (1), and their incidence—particularly that of acetabular fractures in elderly patients—appears to be increasing (2). Despite advances in surgical techniques, achieving adequate mechanical stability while minimizing surgical invasiveness in complex fracture patterns remains challenging.

For displaced acetabular fractures, open reduction and internal fixation (ORIF) remains the standard treatment, while percutaneous cannulated screw techniques are increasingly applied in selected cases to reduce surgical morbidity (3). Current fixation strategies mainly include plate fixation, cannulated screw fixation, and combined screw–plate constructs; however, their relative advantages vary across fracture morphologies and functional demands. Biomechanical and finite-element studies further demonstrate that construct performance is highly dependent on loading conditions, with notable differences observed between standing and sitting scenarios, complicating the translation of single-condition findings into clinical decision-making. Although minimally invasive, image-guided screw placement has gained popularity for reducing soft-tissue disruption, it is constrained by narrow and anatomically variable osseous corridors, a high dependence on precise trajectory control, and the risk of screw malposition under conventional fluoroscopy (4–6). These limitations have driven the adoption of intraoperative 3D imaging and navigation technologies, albeit at the cost of increased workflow complexity and radiation or technology-related trade-offs.

Recent advances in three-dimensional printing have enabled the development of patient-specific drill guides to assist cannulated screw placement in acetabular fracture surgery, aiming to improve trajectory control within narrow and anatomically variable osseous corridors. Clinical application has been reported mainly in technical or case-based studies, including the use of a 3D-printed personalized drill guide to facilitate posterior column screw insertion in displaced acetabular fractures (7). Experimental and cadaveric investigations further demonstrate that 3D-printed drilling guides, including plate-referenced or implant-associated templates, can achieve high placement accuracy for posterior column or infra-acetabular screws while reducing the risk of cortical or intra-articular breach (8, 9). Nevertheless, the available evidence is largely limited to case reports and cadaveric studies, focusing primarily on technical feasibility and placement accuracy, and the real-world perioperative and functional benefits of guide-assisted cannulated screw fixation in acetabular fractures remain insufficiently established.

Accordingly, the aim of this study was to compare perioperative outcomes among three internal fixation strategies—3D-printed guide–assisted cannulated screw plus plate fixation, conventional cannulated screw plus plate fixation, and plate-only fixation—and to complement the clinical findings with finite element analyses understanding and sitting conditions. Through this integrated clinical–biomechanical evaluation, we sought to provide practical evidence to support fixation strategy selection and surgical planning in complex pelvic and acetabular fractures.

## Materials and methods

### Study design

A combined study design integrating a retrospective clinical analysis and a three-dimensional finite element (FE) biomechanical analysis was adopted. The clinical component compared perioperative outcomes, complications, and short-term functional recovery among different internal fixation strategies for pelvic and acetabular fractures. The FE analysis was conducted to evaluate the biomechanical characteristics of three fixation methods under simulated standing and sitting conditions, providing mechanical support for the clinical findings.

### Patients and clinical data

Patients with pelvic or acetabular fractures treated surgically at our institution between November 2021 and May 2023 were retrospectively reviewed. Inclusion criteria were: (1) Tile B3 or Tile C pelvic fractures, or acetabular fractures involving the anterior column, posterior column, transverse, or complex patterns; and (2) treatment with internal fixation. Exclusion criteria included conservative treatment, severe concomitant visceral injuries, inability to tolerate surgery, and old fractures. According to the internal fixation strategy and surgical instruments used, patients were divided into three groups: the 3D-printed guide plate–assisted cannulated screw plus plate group (3D-printed guide + Screw + Plate group), the cannulated screw plus plate group (Screw + Plate group), and the plate-only group. Perioperative data, postoperative complications, and functional outcomes were collected for analysis.

### Surgical technique and three-dimensional assistance

All patients underwent preoperative pelvic CT scanning, and DICOM data were used to generate 1:1 three-dimensional pelvic models for fracture evaluation and surgical planning. The fixation strategy was selected according to fracture type, anatomical location, fracture morphology, and intraoperative stability. Conventional fixation strategies, including plate fixation and cannulated screw fixation, were applied based on fracture characteristics and surgical indications. In selected cases, patients voluntarily chose surgery assisted by a 3D-printed guide after detailed preoperative discussion. All surgeries were performed by the senior orthopedic surgeon, Dr. Zhiping Zhou, together with two assistant surgeons from the same department, thereby helping to maintain consistency in surgical technique and minimize inter-surgeon variability. Surgical procedures were most commonly performed using a Kocher–Langenbeck approach. After fracture exposure and debridement, traction was applied using a Schanz pin placed in the greater trochanter, followed by fracture reduction and temporary fixation with Kirschner wires. Definitive fixation was then achieved using cannulated screws, plates, or their combination according to fracture morphology and intraoperative stability. Fluoroscopy was routinely used to confirm reduction quality and implant positioning.

### Finite element model construction

High-resolution pelvic CT data were obtained from a healthy adult male volunteer (30 years old, 172 cm, 70 kg) without pelvic pathology. CT scanning was performed using a 64-slice dual-source spiral CT scanner with a slice thickness and reconstruction interval of 0.6 mm, and images were exported in DICOM format. The images were imported into Mimics 19.0 for segmentation of bony structures using a threshold range of 220–1,900 HU, followed by manual refinement. The initial triangular mesh models were optimized in 3-matic to reduce surface noise and repair geometric defects. Subsequently, Geomagic Studio 12.0 was used for NURBS surface reconstruction to generate smooth, continuous, CAD-compatible pelvic models.

**Figure 1:**
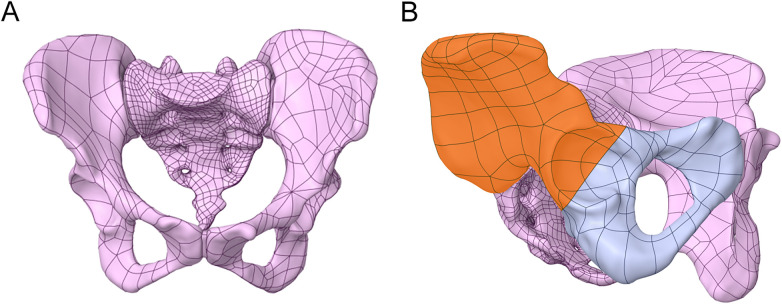
Construction of the finite element model of the pelvis and acetabular fracture. **(A)** Three-dimensional pelvic finite element mesh generated from CT data, illustrating the discretized intact pelvic geometry used for model development. **(B)** Finite element model of a representative both-column (“floating”) acetabular fracture, with the anterior and posterior column fragments segmented and color-coded to define fracture morphology and subsequent fixation modeling.

**Figure 2:**
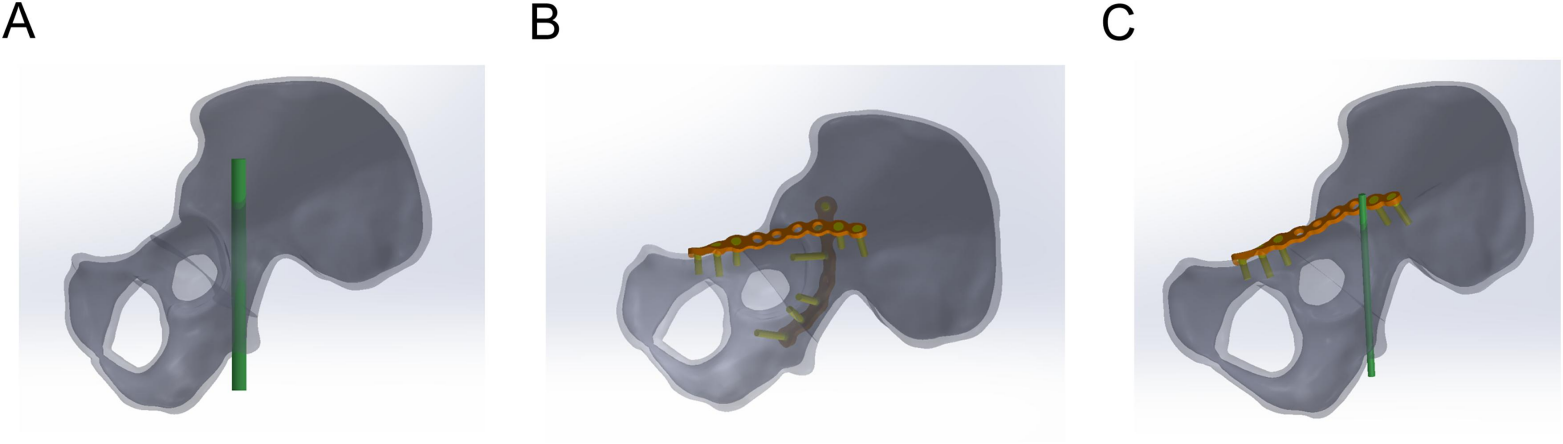
Finite element models of a representative botH,column (“floating”) acetabular fracture with different internal fixation strategies. **(A)** Cannulated screw–only fixation model, simulating percutaneous cannulated screw fixation along the posterior column. **(B)** Plate-only fixation model, representing reconstruction plate fixation applied to the acetabular column without cannulated screw assistance. **(C)** Combined fixation model, consisting of 3D-printed guide plate–assisted cannulated screw placement combined with plate fixation. All models were constructed based on identical fracture geometry and implant dimensions and were used for subsequent finite element analyses under standardized loading conditions.

Cortical and cancellous bone were differentiated by offsetting the outer cortical surface inward by 1.5 mm, with the shell defined as cortical bone and the internal volume as cancellous bone. Articular cartilage layers with uniform thickness were constructed at the sacroiliac joints and pubic symphysis. A representative both-column acetabular fracture (floating acetabulum) was simulated based on clinically common fracture lines (Figure 1). Three fixation configurations—cannulated screw fixation, plate combined with cannulated screw fixation, and plate fixation alone—were modeled in SolidWorks 2017 according to actual implant dimensions and assembled following standard surgical principles (Figure 2).

### Mesh generation and material properties

The complete assembly, including bone, cartilage, ligaments, and fixation devices, was imported into ANSYS 2019 R1 for meshing and analysis. High-order tetrahedral elements were used for all components. A global mesh size of 1.5 mm was applied, with local refinement to 1.0 mm around fracture lines and screw–bone interfaces (Table 1). All materials were assumed to be homogeneous, isotropic, and linearly elastic, and elastic modulus and Poisson's ratio were assigned according to published biomechanical literature (Table 2). Major pelvic ligaments, including the anterior sacroiliac ligament, posterior long and short sacroiliac ligaments, sacrospinous ligament, and sacrotuberous ligament, were simulated using nonlinear spring elements. Ligament attachment points were defined based on anatomical landmarks, and stiffness values were derived from previous experimental studies.

**Table 1:** Mesh information of finite element models.

Fixation method	Number of elements	Number of nodes
Cannulated screw only	563,506	964,523
Cannulated screw + plate	568,983	972,044
Plate only	573,242	980,333

**Table 2:** Material properties assigned in the finite element models.

Material	Elastic modulus (MPa)	Poisson's ratio
Cortical bone (sacrum)	17,000	0.30
Cortical bone (ilium)	6,140	0.30
Cancellous bone (sacrum)	132	0.20
Cancellous bone (ilium)	1,400	0.30
Interpubic disc	5	0.45
Articular cartilage	100	0.30
Titanium alloy plate	110,000	0.30
Titanium alloy screw	110,000	0.30

### Boundary conditions and loading

Contact between fracture surfaces was defined as frictional contact with a friction coefficient of 0.1, while screw–bone interfaces were defined as bonded. Two physiological loading conditions were simulated. In the standing position, all degrees of freedom at the bilateral sacroiliac joints were fully constrained, and a vertical load of 700 N was evenly applied to both acetabular surfaces to represent body weight. In the sitting position, all degrees of freedom at the bilateral ischial tuberosities were constrained, and a vertical load of 700 N was applied to the superior surface of the sacrum. Model validity was assessed by analyzing stress transmission patterns in the intact pelvis understanding conditions and comparing them with established pelvic biomechanics theory and previously published finite element studies.

### Outcome measures

For the finite-element analysis, outcome measures included the stress and displacement distribution of the pelvic structure, as well as the mechanical behavior of the internal fixation devices. Specifically, the maximum von Mises stress and displacement at the acetabular fracture line were recorded for each fixation configuration. In addition, eight measurement points were selected along the fracture line at 5-mm intervals, and the stress and displacement values at each point were extracted to calculate the mean values and standard deviations. Stress distribution, maximum stress, and overall deformation of the fixation devices were also evaluated to assess implant stability under different loading conditions.

### Statistical analysis

Statistical analysis was performed using SPSS software (version 23.0; SPSS Inc., Chicago, IL, USA). Continuous variables were first assessed for normality using the Shapiro–Wilk test and for homogeneity of variance using Levene's test. Variables that satisfied both normality and homogeneity of variance assumptions were compared among the three groups using one-way analysis of variance (ANOVA). Variables that did not meet the assumptions of normal distribution or homogeneity of variance were analyzed using the Kruskal–Wallis test. When the overall ANOVA result was significant, Tukey's honestly significant difference (HSD) test was used for *post hoc* pairwise comparisons. When the Kruskal–Wallis test showed a significant overall difference, Mann–Whitney U tests were performed for pairwise comparisons with Bonferroni correction to adjust the *P* values. Categorical variables were compared using the *χ*^2^ test or Fisher's exact test, as appropriate.

To further evaluate the independent association between fixation strategy and clinical outcomes, multivariable regression analyses were performed. For continuous outcomes, multivariable linear regression models were used. For categorical variables, multivariable logistic regression analysis was performed. The plate-only group was used as the reference group for comparisons, and additional pairwise comparisons between the 3D-printed guide group and the conventional screw plus plate group were also conducted. Covariates included age, sex, polytrauma, shock, comminuted fracture, preoperative hemoglobin, and preoperative D-dimer level. Regression coefficients or odds ratios with 95% confidence intervals were reported as appropriate. All statistical analyses were two-sided, and *P* < 0.05 was considered statistically significant.

## Results

### Pelvic stress distribution

**Figure 3:**
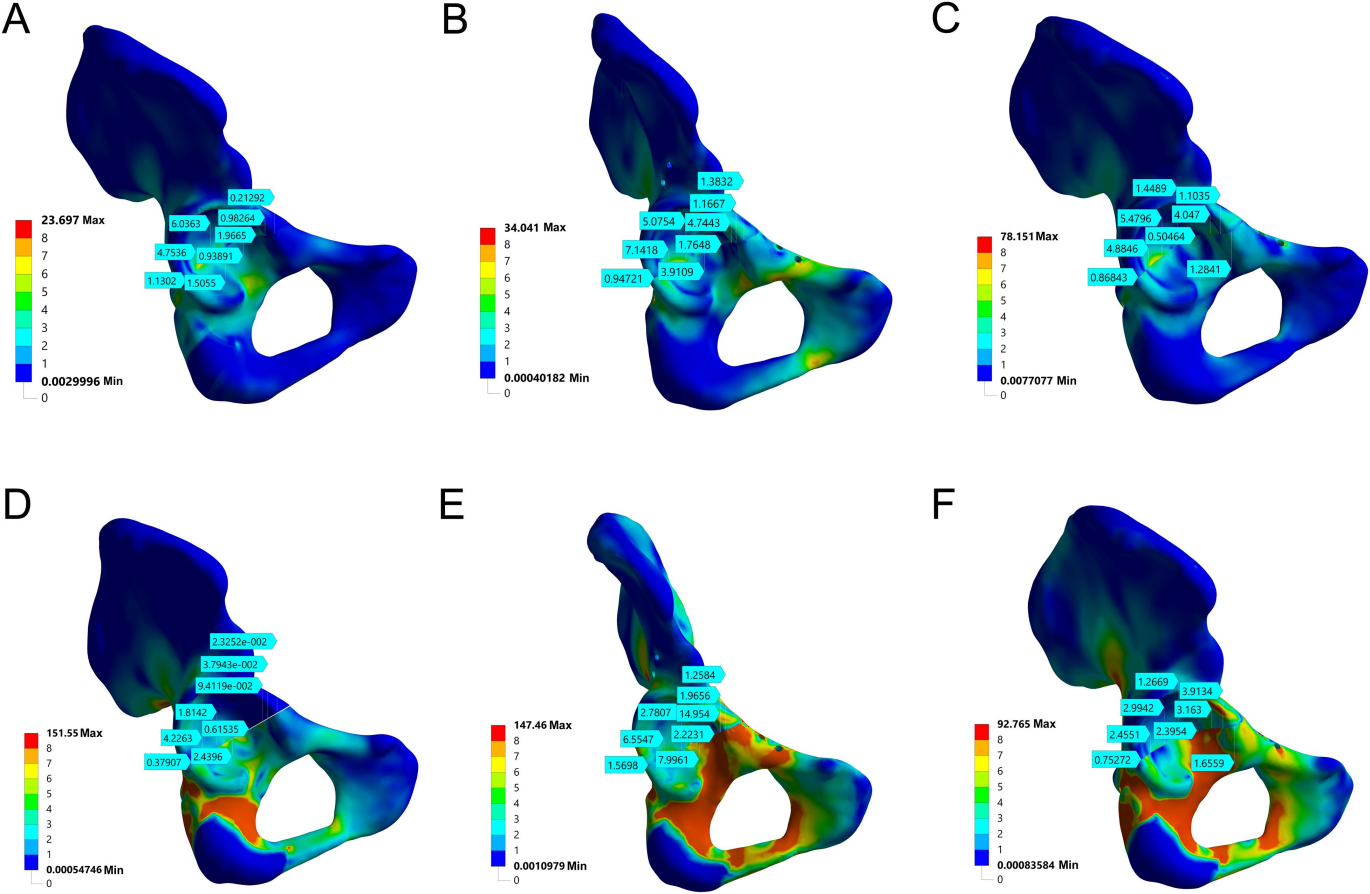
Von mises stress distribution of the pelvic finite element models under different fixation strategies and loading conditions. Stress distribution under the standing condition for the cannulated screw–only fixation model **(A)**, the plate-only fixation model **(B)**, and the combined cannulated screw and plate fixation model **(C)** Stress distribution under the sitting condition for the cannulated screw–only fixation model **(D)**, the plate-only fixation model **(E)**, and the combined cannulated screw and plate fixation model **(F)** All models were analyzed using identical material properties, boundary conditions, and loading protocols, and stress values are displayed using a consistent color scale.

The pelvic von Mises stress contours demonstrated clear differences among fixation configurations under both postures. Under the standing condition, stress was mainly distributed around the periacetabular region and along the pelvic brim. The cannulated screw–only model (Figure 3A) showed the lowest peak pelvic stress (23.697 MPa). The plate-only model (Figure 3B) exhibited a higher peak stress (34.041 MPa) with more pronounced stress concentration around the acetabular region. The cannulated screw plus plate model (Figure 3C) presented the highest peak pelvic stress among the standing simulations (78.151 MPa), with a focal high-stress region adjacent to the acetabulum. Under the sitting condition, pelvic stress increased markedly in all models compared with the standing condition, and high-stress regions extended toward the inferior pelvic ring. The cannulated screw–only model (Figure 3D) reached the highest peak pelvic stress (151.55 MPa), followed by the plate-only model (Figure 3E) (147.46 MPa). In contrast, the cannulated screw plus plate model (Figure 3F) showed a comparatively lower peak stress (92.765 MPa). Overall, the sitting condition resulted in higher peak pelvic stress than the standing condition across all fixation methods, with posture-dependent variations in both stress magnitude and spatial distribution.

### Stress and displacement at the acetabular fracture line

Quantitative analysis of stress and displacement at the acetabular fracture line revealed distinct differences among the three fixation methods under both standing and sitting conditions (Table 3). In the standing position, the plate-only group exhibited the highest mean fracture-line stress (3.2794 ± 2.2508 MPa) and maximum stress (8.4466 MPa), followed by the cannulated screw plus plate group (mean stress: 2.5823 ± 1.9701 MPa; maximum stress: 7.2931 MPa). The cannulated screw–only group demonstrated the lowest mean (2.1908 ± 2.0142 MPa) and maximum stress (7.5657 MPa). A similar trend was observed for displacement, with the plate-only group showing the greatest mean (0.4246 ± 0.0528 mm) and maximum displacement (0.50929 mm), whereas the cannulated screw–only group showed the smallest displacement values. Under the sitting condition, fracture-line stress increased in all fixation models compared with standing. The plate-only group demonstrated the highest mean stress (4.9105 ± 4.8237 MPa) and a markedly elevated maximum stress of 15.36 MPa. The cannulated screw plus plate group showed intermediate stress values (mean: 2.5743 ± 1.0678 MPa; maximum: 5.4321 MPa), while the cannulated screw–only group exhibited the lowest mean (1.2037 ± 1.5786 MPa) and maximum stress (4.6085 MPa). In terms of displacement, the cannulated screw plus plate group showed the smallest mean (0.1257 ± 0.0754 mm) and maximum displacement (0.26362 mm), whereas the cannulated screw–only group demonstrated the largest maximum displacement (0.62815 mm) under sitting conditions.

**Table 3:** Detailed comparison of baseline characteristics and clinical outcomes.

Variable	3D-printed Guide + Screw + Plate (A) (*n* = 8)	Screw + Plate (B) (*n* = 14)	Plate Only (C) (*n* = 12)	*Overall P*-value	Pairwise *P*-value (<0.05)
Demographics and Clinical Characteristics					
Age (years)	43.75 ± 11.32	54.57 ± 10.32	53.25 ± 16.25	0.161	-
Gender (Male), *n* (%)	5 (62.5%)	7 (50.0%)	9 (75.0%)	0.675	-
Polytrauma, *n* (%)	3 (37.5%)	4 (28.6%)	3 (25.0%)	0.793	-
Shock, *n* (%)	1 (12.5%)	0 (0.0%)	1 (8.3%)	0.491	-
Comminuted Fracture, *n* (%)	0 (0.0%)	2 (14.3%)	2 (16.7%)	0.467	-
Pre-op Hb (g/L)	93.62 ± 8.88	104.50 ± 18.75	99.00 ± 13.99	0.281	-
Pre-op D-dimer (mg/L)	11.28 ± 4.30	5.41 ± 4.11	9.03 ± 5.26	**0**.**006**	**A vs. B: 0.011**
**Perioperative Outcomes**					-
Operative time (min)	144.75 ± 48.78	167.29 ± 65.60	174.92 ± 43.04	0.316	-
Blood loss (mL)	360.62 ± 334.39	425.36 ± 247.45	870.83 ± 384.03	**0**.**002**	**B vs. C: 0.004** **A vs. C: 0.031**
Incision length (cm)	10.69 ± 6.78	14.64 ± 1.69	20.33 ± 7.39	**0**.**002**	**A vs. B: 0.033** **B vs. C: 0.036** **A vs. C: 0.028**
Number of incisions	1.25 ± 0.46	1.29 ± 0.47	1.33 ± 0.65	0.985	-
**Postoperative Outcomes**					-
3-month Hip Score	86.00 ± 12.82	84.36 ± 5.43	85.50 ± 6.26	0.184	-
Complications, *n* (%)	4 (50.0%)	3 (21.4%)	5 (41.7%)	0.245	-
**Health Economics**					-
Hospital stay (days)	31.88 ± 8.27	24.00 ± 16.32	33.67 ± 26.56	**0**.**031**	-
Total Cost (RMB)	85,984.69 ± 58,439.34	53,795.16 ± 28,855.73	1,05,016.60 ± 57,086.16	**0**.**033**	**B vs. C: 0.027**

Data are presented as mean ± standard deviation or *n* (%). Statistical significance was set at *P* < 0.05 (bolded). Continuous variables were analyzed using One-way ANOVA or Kruskal–Wallis H test, depending on the normality and homogeneity of variance. *post-hoc* pairwise comparisons were conducted using Tukey's HSD test or Mann–Whitney U test with Bonferroni correction. Pearson's chi-square test or Fisher's exact test were used for categorical variables. 3D, three-dimensional; Hb, hemoglobin; RMB, Renminbi; Pre-op, preoperative.

### Biomechanical performance of fixation devices

**Figure 4:**
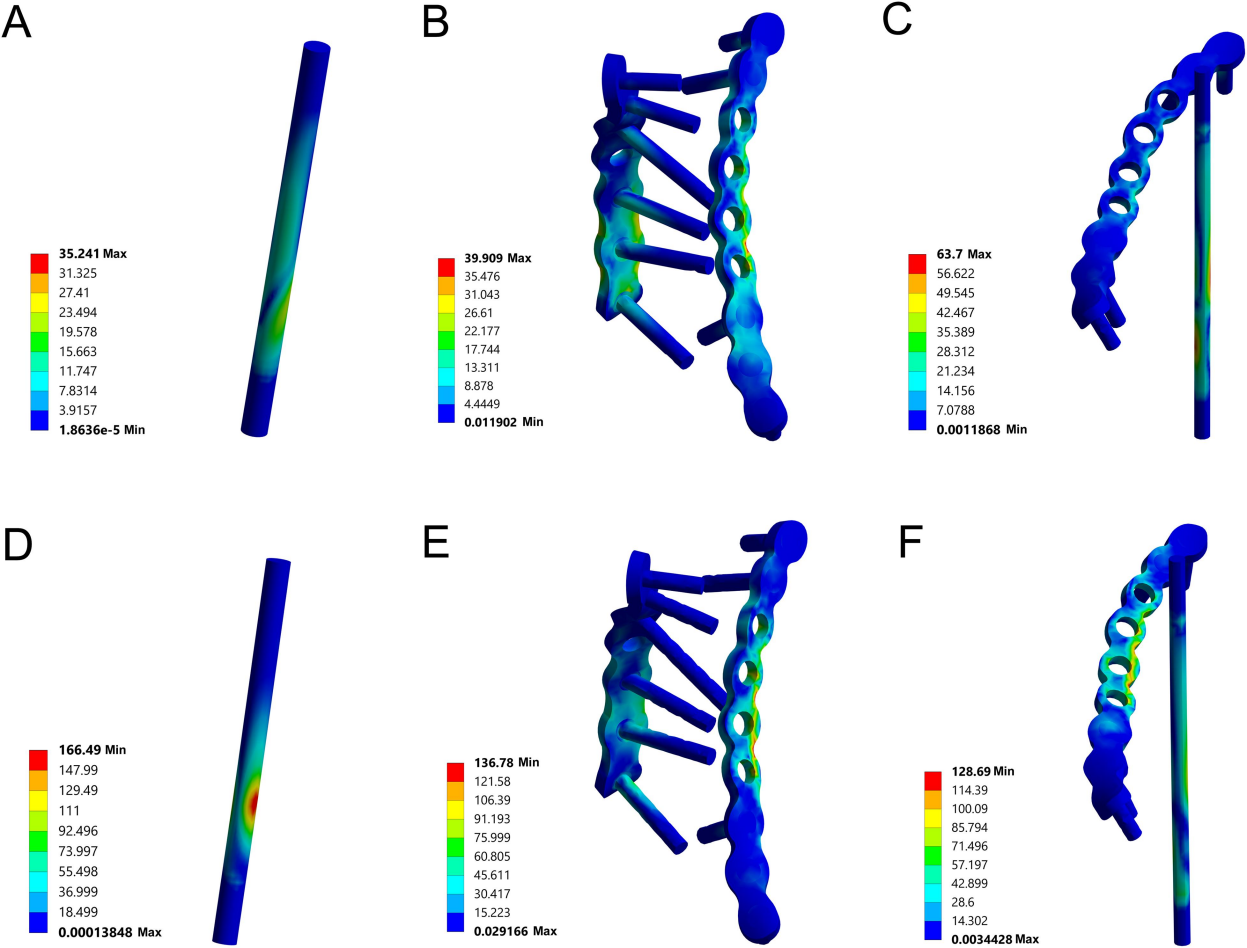
Von mises stress distribution of internal fixation devices in the finite element models under different fixation configurations and loading conditions. Stress distribution under the standing condition for the cannulated screw–only fixation model **(A)**, the plate-only fixation model **(B)**, and the combined cannulated screw and plate fixation model **(C)** Stress distribution under the sitting condition for the cannulated screw–only fixation model **(D)**, the plate-only fixation model **(E)**, and the combined cannulated screw and plate fixation model **(F)** All fixation devices were modeled with identical material properties and implant geometries and analyzed under standardized boundary conditions and loading protocols.

Von Mises stress distributions differed across the three fixation methods under both loading conditions (Figures 4A–F). Stress was primarily concentrated along the cannulated screw shaft and at screw–plate junctions in plate-based constructs, whereas a more uniform distribution was observed in the cannulated screw–only model. Although peak stress magnitude and location varied by fixation type and posture, stress concentrations remained localized.

As shown in Table 4, fixation methods differed in peak stress and displacement. Understanding conditions, the cannulated screw plus plate group exhibited the highest maximum stress (63.7 MPa), followed by the plate-only (39.91 MPa) and cannulated screw–only groups (35.24 MPa). Under sitting conditions, peak stress increased in all models, with the cannulated screw–only group showing the highest value (166.49 MPa), followed by the plate-only (136.78 MPa) and cannulated screw plus plate groups (128.69 MPa). All values were well below the elastic modulus of the titanium alloy (110,000 MPa). Regarding displacement, the plate-only group showed the largest displacement understanding conditions (0.55608 mm), while the cannulated screw–only group showed the smallest (0.33095 mm). Under sitting conditions, displacement increased most in the cannulated screw–only group (0.82323 mm), whereas the cannulated screw plus plate group demonstrated the smallest displacement (0.36274 mm).

**Table 4:** Multivariate analysis of perioperative and functional outcomes among different internal fixation methods.

Outcome Variable	Comparison groups	Adjusted Difference (β/ OR)^a^	95% CI	*P* value
Primary outcomes				
Operation Time (min)	3D-printed Guide vs. Plate	−56.15	−111.76 to −0.54	**0**.**048**
	Cannulated Screw + Plate vs. Plate	12.32	−35.10–59.73	0.597
	3D-printed Guide vs. Cannulated Screw + Plate	−68.46	−130.19 to −6.73	**0**.**031**
Intraoperative Blood Loss (mL)	3D-printed Guide vs. Plate	−575.48	−899.17 to −251.79	**0**.**001**
	Cannulated Screw + Plate vs. Plate	−354.26	−630.23 to −78.30	**0**.**014**
	3D-printed Guide vs. Cannulated Screw + Plate	−221.22	−580.52 to 138.08	0.216
Total Incision Length (cm)	3D-printed Guide vs. Plate	−7.94	−13.10 to −2.78	**0**.**004**
	Cannulated Screw + Plate vs. Plate	−5.05	−9.45 to −0.65	**0**.**026**
	3D-printed Guide vs. Cannulated Screw + Plate	−2.89	−8.63–2.84	0.308
** **3-Month Hip Function Score	3D-printed Guide vs. Plate	−0.66	−8.51–7.19	0.864
	Cannulated Screw + Plate vs. Plate	−0.70	−7.39–5.99	0.831
	3D-printed Guide vs. Cannulated Screw + Plate	0.04	−8.67–8.75	0.993
Complications	3D-printed Guide vs. Plate	1.72	0.13–21.92	0.677
	Cannulated Screw + Plate vs. Plate	0.31	0.03–3.21	0.326
	3D-printed Guide vs. Cannulated Screw + Plate	5.55	0.30–101.52	0.248
**Secondary outcomes**				
Hospital stay (days)	3D-printed Guide vs. Plate	−2.44	−20.92–16.05	0.788
	Cannulated Screw + Plate vs. Plate	−9.74	−25.50–6.02	0.214
	3D-printed Guide vs. Cannulated Screw + Plate	7.3	−13.21–27.82	0.47
Total Cost (RMB)	3D-printed Guide vs. Plate	−39944.29	−77808.26 to −2080.32	**0**.**040**
	Cannulated Screw + Plate vs. Plate	−32527.55	−64809.10 to −246.00	**0**.**048**
	3D-printed Guide vs. Cannulated Screw + Plate	−7416.74	−49446.64–34613.17	0.719

CI, Confidence Interval. Adjusted covariates included Age, Gender, Polytrauma, Shock, Comminuted Fracture, Preoperative Hemoglobin (Hb), and Preoperative D-dimer^2^.

a For the “Complications” outcome, the value represents the Odds Ratio (OR); for other continuous variables, it represents the regression coefficient. *P* < 0.05 indicates statistical significance.

Bold numbers represent *P* value less than 0.05.

### Baseline demographic and clinical characteristics

A total of 34 patients met the inclusion criteria, including 21 males and 13 females, with a mean age ranging from 43.75 to 54.57 years across groups. According to the internal fixation strategy, patients were allocated to the 3D-printed surgical guide plate–assisted screw plus plate group (*n* = 8), the screw plus plate group (*n* = 14), or the plate-only group (*n* = 12). All patients successfully underwent surgery and completed at least 3 months of follow-up. Baseline demographic and clinical characteristics are summarized in Table 3.

No statistically significant differences were observed among the three groups in terms of age (*P* = 0.171) or sex distribution (*P* = 0.675). Similarly, injury-related variables, including polytrauma (*P* = 0.793), hemorrhagic shock (*P* = 0.491), and comminuted fractures (*P* = 0.467), were comparable across groups. Preoperative hemoglobin levels did not differ significantly among groups (*P* = 0.353). However, a significant difference was observed in preoperative D-dimer levels (*P* = 0.013), with higher values noted in the 3D-printed guide–assisted group. Variables demonstrating potential baseline imbalance were therefore adjusted for in subsequent multivariate analyses.

**Figure 5:**
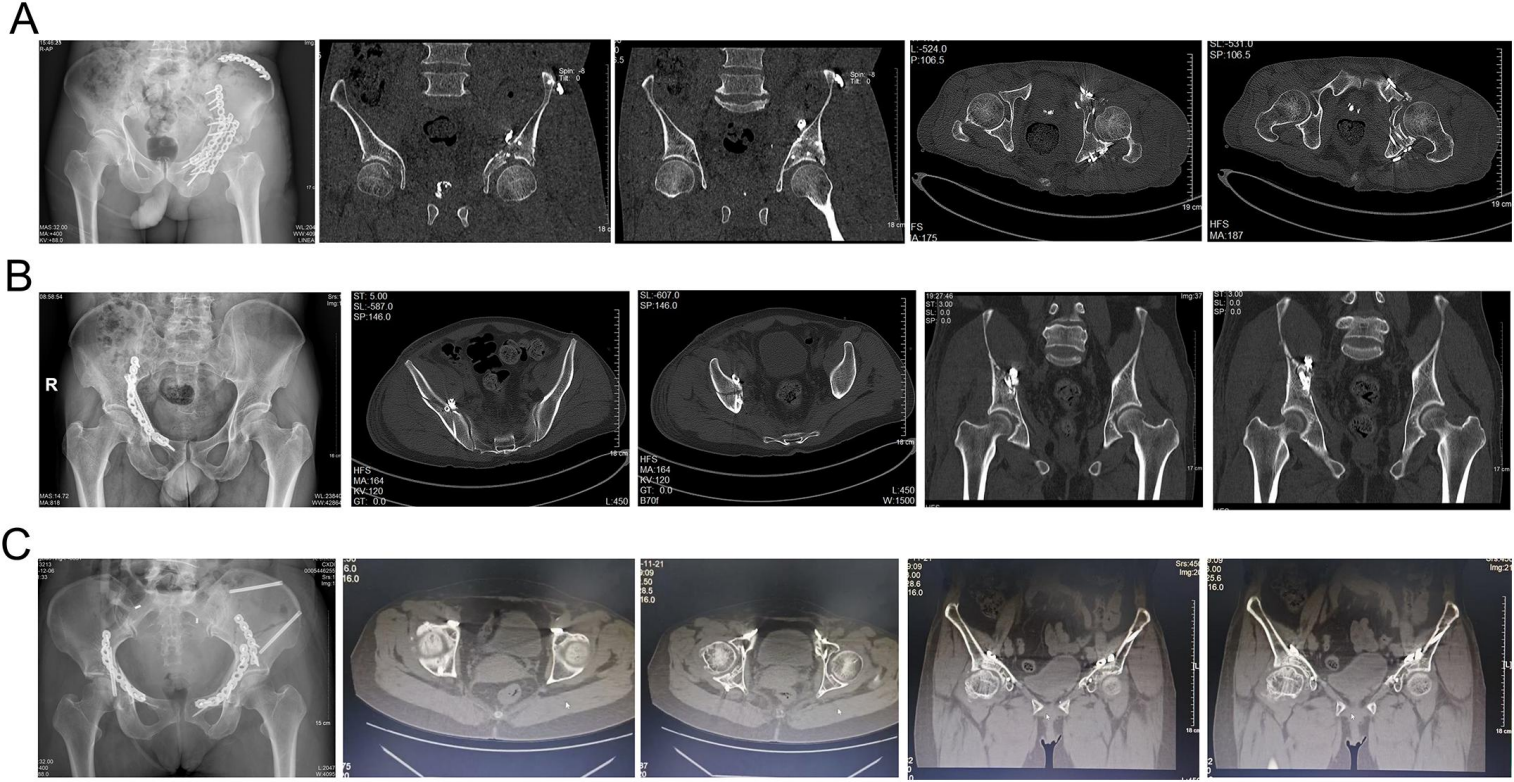
Representative postoperative radiographic and computed tomography (CT) images of the three fixation strategies. **(A)** Plate-only fixation group. **(B)** Cannulated screw plus plate fixation group. **(C)** 3D-printed guide–assisted cannulated screw plus plate fixation group. Anteroposterior pelvic radiographs and postoperative axial and coronal CT images are shown for each group.

Representative postoperative radiographic and CT images of the three fixation strategies are shown in Figure 5. In the plate-only group, postoperative imaging demonstrated satisfactory fracture reduction and stable fixation using multiple reconstruction plates. In the cannulated screw plus plate group, combined fixation with cannulated screws and plates achieved acceptable fracture alignment and reliable implant positioning. In the 3D-printed guide–assisted cannulated screw plus plate group, postoperative CT images showed satisfactory fracture reduction and accurate cannulated screw placement without obvious intra-articular penetration. Overall, postoperative imaging confirmed satisfactory implant positioning and maintenance of fracture alignment in all three groups.

### Primary outcomes: perioperative indicators, safety, and functional recovery

Univariate analysis of baseline demographic and clinical characteristics (Table 3) showed that age, sex distribution, polytrauma, shock, comminuted fracture, and preoperative hemoglobin levels were comparable among the three groups (all *P* > 0.05). However, preoperative D-dimer levels differed significantly among groups (*P* = 0.006), with pairwise comparison showing a higher level in the 3D-printed guide + screw + plate group than in the screw + plate group (11.28 ± 4.30 mg/L vs. 5.41 ± 4.11 mg/L; *P* = 0.011).

Regarding perioperative outcomes, operative time was numerically shortest in the 3D-printed guide + screw + plate group, followed by the screw + plate group and the plate-only group, but the difference was not statistically significant (144.75 ± 48.78 min vs. 167.29 ± 65.60 min vs. 174.92 ± 43.04 min; *P* = 0.316). Intraoperative blood loss differed significantly among the three groups (*P* = 0.002). Pairwise comparisons showed that blood loss was significantly lower in both the 3D-printed guide + screw + plate group and the screw + plate group than in the plate-only group (360.62 ± 334.39 mL vs. 870.83 ± 384.03 mL, *P* = 0.031; 425.36 ± 247.45 mL vs. 870.83 ± 384.03 mL, *P* = 0.004, respectively), whereas no significant difference was observed between the 3D-printed guide + screw + plate group and the screw + plate group. Incision length also differed significantly among groups (*P* = 0.002). Pairwise comparisons showed that the 3D-printed guide + screw + plate group had a shorter incision length than both the screw + plate group and the plate-only group (10.69 ± 6.78 cm vs. 14.64 ± 1.69 cm, *P* = 0.033; 10.69 ± 6.78 cm vs. 20.33 ± 7.39 cm, *P* = 0.028, respectively), while the screw + plate group also had a shorter incision length than the plate-only group (14.64 ± 1.69 cm vs. 20.33 ± 7.39 cm; *P* = 0.036). The number of incisions was similar among the three groups, with no statistically significant difference (1.25 ± 0.46 vs. 1.29 ± 0.47 vs. 1.33 ± 0.65; *P* = 0.985).

For postoperative outcomes, the 3-month hip score was comparable among the three groups (86.00 ± 12.82 vs. 84.36 ± 5.43 vs. 85.50 ± 6.26; *P* = 0.184), indicating similar short-term functional recovery across fixation strategies. Postoperative complication rates were 50.0%, 21.4%, and 41.7% in the 3D-printed guide + screw + plate group, screw + plate group, and plate-only group, respectively, with no statistically significant difference among groups (*P* = 0.245). Specifically, in the 3D-printed guide + screw + plate group, postoperative complications occurred in 4 of 8 patients, including lower extremity deep venous thrombosis in 2 patients, incision fat liquefaction with delayed wound healing in 1 patient, and pulmonary infection in 1 patient. In the screw + plate group, complications occurred in 3 of 14 patients, including lower extremity deep venous thrombosis in 1 patient, pulmonary infection in 1 patient, and urinary tract infection in 1 patient. In the plate-only group, complications occurred in 5 of 12 patients, including poor incision healing in 2 patients, lower extremity deep venous thrombosis in 2 patients, and sciatic nerve injury in 1 patient. All patients improved after appropriate treatment and were discharged successfully.

After adjustment for age, sex, polytrauma, shock, comminuted fracture, preoperative hemoglobin, and D-dimer levels, multivariable analysis demonstrated significant differences in clinical outcomes among fixation strategies (Table 4). Compared with the plate-only group, the 3D-printed guide–assisted group had a significantly shorter operative time (adjusted difference −56.15 min; 95% CI −111.76 to −0.54; *P* = 0.048), significantly reduced intraoperative blood loss (−575.48 mL; 95% CI −899.17 to −251.79; *P* = 0.001), and a shorter total incision length (−7.94 cm; 95% CI −13.10 to −2.78; *P* = 0.004). Operative time was also significantly shorter in the 3D-printed guide group compared with the cannulated screw plus plate group (−68.46 min; 95% CI −130.19 to −6.73; *P* = 0.031). The cannulated screw plus plate group showed significantly reduced intraoperative blood loss (−354.26 mL; 95% CI −630.23 to −78.30; *P* = 0.014) and shorter incision length (−5.05 cm; 95% CI −9.45 to −0.65; *P* = 0.026) compared with the plate-only group, whereas operative time did not differ significantly between these two groups (*P* = 0.597). No significant adjusted differences were observed among the three groups in 3-month hip function scores (all *P* > 0.80) or complication risk (all *P* > 0.24).

### Secondary outcomes: hospital stay and health economics

For secondary outcomes, univariate analysis revealed hospital stay differed significantly among the three groups (31.88 ± 8.27 days vs. 24.00 ± 16.32 days vs. 33.67 ± 26.56 days; *P* = 0.031), although no significant pairwise difference was identified. Total hospitalization cost also differed significantly among groups (*P* = 0.033), with the screw + plate group showing a significantly lower total cost than the plate-only group (53,795.16 ± 28,855.73 RMB vs. 105,016.60 ± 57,086.16 RMB; *P* = 0.027) (Table 3). After multivariate adjustment, hospital length of stay remained comparable across groups (all *P* > 0.05). In contrast, total hospitalization cost remained significantly lower in both the 3D-printed guide group and the Screw + Plate group compared with the Plate-only group. Specifically, the adjusted cost reduction was 39,944.29 RMB for the 3D-printed guide group (95% CI: −77,808.26 – −2,080.32; *P* = 0.040) and 32,527.55 RMB for the Screw + Plate group (95% CI: −64,809.10 – −246.00; *P* = 0.048). No significant difference in total cost was observed between the 3D-printed guide group and the Screw + Plate group (adjusted difference: −7,416.74 RMB; 95% CI: −49,446.64–34,613.17; *P* = 0.719) (Table 4).

## Discussion

This study shows that 3D-printed guide–assisted cannulated screw combined with plate fixation is associated with reduced operative time, blood loss, and surgical exposure, while maintaining comparable short-term complication rates and functional outcomes to conventional fixation strategies. Finite element analysis demonstrated pronounced posture-dependent biomechanical differences, with all constructs experiencing higher stress and displacement under sitting than standing conditions. Importantly, combined screw–plate fixation provided more effective fracture-line displacement control under sitting loads, whereas cannulated screw–only fixation exhibited lower stress in selected scenarios but reduced overall stability. Together, these findings highlight the clinical value of guide-assisted minimally invasive fixation and emphasize the need to incorporate posture-related biomechanical considerations into fixation strategy selection for complex pelvic and acetabular fractures.

Our perioperative findings are broadly consistent with the growing body of evidence supporting three-dimensional (3D) printing–assisted workflows in pelvic and acetabular trauma. In a meta-analysis focusing on acetabular fracture surgery, 3D printing assistance (predominantly for preoperative planning and plate precontouring) was associated with a mean reduction in operative time of 38.8 min and intraoperative blood loss of 259.7 mL (both statistically significant), alongside shorter instrumentation time, compared with conventional techniques (10). Expanding beyond acetabular fractures alone, a more recent systematic review/meta-analysis of pelvic and acetabular fractures reported similar advantages using pooled effect estimates (e.g., ratio of means for operative duration 0.74 and for blood loss 0.71), with fewer intraoperative imaging events (odds ratio 0.36) and lower complication odds (odds ratio 0.42), while also increasing the likelihood of excellent/good reduction (odds ratio 1.53) (11). Importantly, however, much of this literature evaluates “3D printing” as an aid to visualization and implant preparation rather than as a true intraoperative trajectory-control solution. In this context, cadaveric studies of patient-specific or plate-referenced drill guides provide more direct evidence for accurate and safe screw insertion in narrow osseous corridors: Meesters et al. reported small median deviations between planned and achieved screw directions (approximately 5.9°–7.6°) and entry points (about 2.6–3.6 mm), with no intra-articular penetration (9), while Freigang et al. achieved corridor-contained infra-acetabular screw placement in all specimens without intra-articular or intrapelvic breach (8). These guide-based data complement, but do not replace, navigation evidence—classic comparative work suggested that 3D-fluoroscopy navigation can reduce cortical/articular perforation (7% vs. 20%) at the cost of longer procedure/fluoroscopy times and higher radiation dose (12). Taken together, the existing literature supports perioperative efficiency gains from 3D-assisted strategies and demonstrates high placement accuracy for guide-based screw insertion, yet direct clinical comparisons among combined fixation constructs and studies integrating posture-specific biomechanical validation remain limited—an evidence gap that the present clinical-plus-FE framework is designed to address.

3D-printed guides have been shown to significantly improve screw placement accuracy in pelvic and acetabular fractures, especially in complex fractures involving narrow anatomical corridors. Studies demonstrate that these guides reduce screw position deviations, particularly in challenging insertions like posterior column screws, achieving accuracy within 1–1.3 mm compared to traditional free-hand methods. One cadaveric study confirmed that 3D-printed guides resulted in more precise screw placements with minimal deviation, thus improving surgical outcomes in difficult cases (13). Clinical data further supports these findings, showing that 3D-printed guides contribute to a reduction in operative time, with an average decrease of 30–60 min, along with lower blood loss and reduced reliance on fluoroscopy, ultimately enhancing surgical efficiency and patient safety. Additionally, the use of 3D guides has been linked to fewer intraoperative complications and a reduction in postoperative malreduction and soft tissue injuries, attributed to the precise screw placements enabled by the guides (10). Furthermore, 3D-printed guides offer a simpler and more intuitive alternative to traditional navigation systems, reducing the dependence on advanced imaging and extensive surgeon experience, which is particularly beneficial for less experienced surgeons. This has been corroborated by multiple studies highlighting the ease of use and accuracy achieved with these patient-specific guides, even in cases requiring complex screw trajectories (14). However, challenges remain, including the high costs of 3D printing technology, the technical complexity of designing individualized guides, and the time required to fabricate these guides, which may limit their use in urgent or emergency settings (15).

Our finite element analysis revealed a clear posture-dependent mechanical response, with sitting generating higher pelvic stress and displacement than standing across all constructs, indicating greater instability risk for acetabular “floating” fractures. This is biomechanically reasonable, as standing loads are mainly transferred through the acetabula to the pelvic ring and sacroiliac complex, whereas sitting shifts load toward the ischial supports, increasing posterior ring demand and shear and bending across fracture planes. Similar posture-related differences in construct behavior have been reported in prior acetabular finite element studies (16), and classical pelvic biomechanics research further confirms that sacroiliac load transfer is highly sensitive to boundary conditions and force closure (17, 18). In our models, screw–plate fixation better controlled fracture-line displacement under sitting loads, while screw-only fixation showed inferior displacement control despite lower fracture-line stress in some scenarios, emphasizing the need to evaluate fixation strategies under posture-specific loading conditions.

Integrating these clinical findings with finite element results, several important implications for surgical decision-making emerge. Patient-specific 3D-printed guides can serve as an effective adjunct to improve surgical efficiency and accuracy while reducing intraoperative invasiveness by facilitating precise screw placement and minimizing repeated adjustments. From a fixation strategy perspective, combined screw–plate fixation appears more suitable for complex fracture patterns or situations associated with higher mechanical loading and instability risk, as it provides superior displacement control and construct stability. In contrast, screw-only fixation should be applied with strict indication selection, as its biomechanical performance may be insufficient in high-load or unstable scenarios despite its minimally invasive nature.

Although no statistically significant difference in complication rates was observed among the groups, the relatively high complication rate in the 3D-printed guide group should be interpreted cautiously. Previous studies have suggested that 3D printing-assisted surgery may facilitate preoperative planning, fracture visualization, implant pre-contouring, screw trajectory design, and intraoperative orientation in acetabular fracture surgery, and meta-analyses have reported potential advantages in operative time, blood loss, fluoroscopy exposure, and complication reduction (19). However, the available evidence remains heterogeneous, and recent clinical studies have indicated that improvements in surgical planning do not necessarily translate into significantly lower postoperative complication rates or superior clinical outcomes (20, 21). In the present study, the observed complications were mainly general perioperative complications, including deep venous thrombosis, pulmonary infection, and wound-healing problems, rather than guide-specific complications such as intra-articular screw penetration, implant malposition, or guide-related neurovascular injury. These findings should be interpreted in the broader context of pelvic and acetabular fracture surgery, in which complications such as venous thromboembolism, infection, wound-healing problems, and nerve injury remain clinically relevant concerns (22, 23). Given the small sample size, this study may have been underpowered to detect clinically meaningful differences in complication risk, and the absence of statistical significance should not be interpreted as evidence of equivalent safety among fixation strategies. Larger prospective studies with standardized complication definitions, stratification by fracture pattern, and longer follow-up are therefore needed to further evaluate the safety profile of 3D-assisted fixation techniques.

It should be emphasized that the finite element analysis in this study was not designed to directly evaluate the biomechanical effect of the 3D-printed guide plate itself. The guide plate is removed after assisting screw trajectory control and does not function as a load-bearing implant after fixation. Therefore, the FE analysis focused on the mechanical behavior of the final implanted constructs, particularly the stability of combined cannulated screw–plate fixation compared with screw-only and plate-only fixation. In this context, the clinical and FE components address complementary aspects of the same treatment strategy: the clinical analysis evaluates whether guide-assisted screw placement improves surgical efficiency and perioperative outcomes, whereas the FE analysis supports the biomechanical rationale for using a combined screw–plate construct in complex acetabular fractures.

This study has several limitations. First, its retrospective, single-center design, small sample size, and short follow-up period may limit external validity and restrict the assessment of long-term functional outcomes, fracture healing, post-traumatic arthritis, implant-related complications, and fixation durability. Although baseline characteristics were generally balanced among groups and potential confounders were adjusted for in the multivariable models, the cohort included heterogeneous pelvic and acetabular fracture patterns, and the limited sample size precluded reliable subgroup analysis according to fracture classification. Therefore, the clinical findings should be interpreted as preliminary evidence rather than definitive proof of superiority for any specific fixation strategy across all fracture subtypes. The small sample size may also have reduced statistical power and affected the stability of the multivariable regression analyses, increasing the risk of overfitting; thus, the discrepancy between the univariable and multivariable findings for operative time should be interpreted cautiously. Second, the finite element model involved several idealized assumptions. Cortical bone, cancellous bone, cartilage, and fixation devices were modeled as homogeneous, isotropic, and linearly elastic materials. Although major pelvic ligaments were simulated using nonlinear spring elements, active pelvic and hip muscle forces were not included, and the bonded screw–bone contact assumption may not fully reproduce *in vivo* interfacial micromotion or partial slipping. Accordingly, the biomechanical results should be interpreted as comparative findings among fixation constructs rather than exact simulations of *in vivo* implant–bone interactions. Future multicenter prospective studies with larger cohorts, longer follow-up, stratified analyses by fracture classification, and more physiologically representative patient-specific finite element models are needed.

## Conclusions

This study demonstrates that combined cannulated screw–plate fixation offers superior mechanical reliability for complex acetabular fractures, while screw-only fixation should be reserved for carefully selected cases. The use of patient-specific 3D-printed guides significantly improves surgical efficiency and reduces operative invasiveness without compromising short-term safety or functional outcomes. These findings support an individualized, biomechanics-informed approach to fixation strategy selection and highlight the clinical value of integrating 3D printing technology into complex acetabular fracture management.

## Data Availability

The raw data supporting the conclusions of this article will be made available by the authors, without undue reservation.
